# Ferroportin mediates the intestinal absorption of iron from a nanoparticulate ferritin core mimetic in mice

**DOI:** 10.1096/fj.14-251520

**Published:** 2014-08

**Authors:** Mohamad F. Aslam, David M. Frazer, Nuno Faria, Sylvaine F. A. Bruggraber, Sarah J. Wilkins, Cornel Mirciov, Jonathan J. Powell, Greg J. Anderson, Dora I. A. Pereira

**Affiliations:** * Medical Research Council Human Nutrition Research, Elsie Widdowson Laboratory, Cambridge, UK;; † Iron Metabolism Laboratory, Queensland Institute of Medical Research Berghofer Medical Research Institute, Brisbane, Queensland, Australia; and; ‡ School of Chemistry and Molecular Bioscience and; § School of Medicine, University of Queensland, Brisbane, Queensland, Australia

**Keywords:** nanoiron, basolateral export, iron homeostasis, hepcidin, knockout mice

## Abstract

The ferritin core is composed of fine nanoparticulate Fe^3+^ oxohydroxide, and we have developed a synthetic mimetic, nanoparticulate Fe^3+^ polyoxohydroxide (nanoFe^3+^). The aim of this study was to determine how dietary iron derived in this fashion is absorbed in the duodenum. Following a 4 wk run-in on an Fe-deficient diet, mice with intestinal-specific disruption of the *Fpn-1* gene (Fpn-KO), or littermate wild-type (WT) controls, were supplemented with Fe^2+^ sulfate (FeSO_4_), nanoFe^3+^, or no added Fe for a further 4 wk. A control group was Fe sufficient throughout. Direct intestinal absorption of nanoFe^3+^ was investigated using isolated duodenal loops. Our data show that FeSO_4_ and nanoFe^3+^ are equally bioavailable in WT mice, and at wk 8 the mean ± sem hemoglobin increase was 18 ± 7 g/L in the FeSO_4_ group and 30 ± 5 g/L in the nanoFe^3+^ group. Oral iron failed to be utilized by Fpn-KO mice and was retained in enterocytes, irrespective of the iron source. In summary, although nanoFe^3+^ is taken up directly by the duodenum its homeostasis is under the normal regulatory control of dietary iron absorption, namely *via* ferroportin-dependent efflux from enterocytes, and thus offers potential as a novel oral iron supplement.—Aslam, M. F., Frazer, D. M., Faria, N., Bruggraber, S. F. A., Wilkins, S. J., Mirciov, C., Powell, J. J., Anderson, G. J., Pereira, D. I. A. Ferroportin mediates the intestinal absorption of iron from a nanoparticulate ferritin core mimetic in mice.

Iron deficiency anemia (IDA) persists as the major nutritional deficiency disorder in the world, affecting >1 billion people (1). This places IDA in the World Health Organization's top 10 list of target diseases for cure and prevention (2–4). Current therapy for IDA involves supplementation with an oral iron preparation. First-generation oral iron agents are simple Fe^2+^ salts, which are cheap and well absorbed, but are associated with significant upper and lower gastrointestinal side effects, such as nausea, constipation, and abdominal discomfort (5, 6). Second-generation oral iron agents are soluble chelated forms of Fe^2+^ or Fe^3+^ and, while they reduce upper gastrointestinal side effects, they are expensive to manufacture and, chronically, they appear to retain their distal gastrointestinal adverse effects (7–9). Indeed, recent studies have consistently shown that soluble oral iron negatively affects the colonic flora, promoting the presence of potentially pathogenic bacteria at the expense of beneficial bacteria (10–13). Other studies have also raised serious concerns over “available” iron in the colon as a risk factor for inflammatory signaling and colorectal carcinogenesis (8, 12).

To help address these issues, we have developed a nanodispersed, ligand-modified material, nanoparticulate Fe^3+^ polyoxohydroxide (nanoFe^3+^). Analysis by high-contrast, high-resolution electron microscopy showed that nanoFe^3+^ has a very similar structure to the Fe^3+^ oxohydroxide core of ferritin (14); that is, it is destabilized 2-line ferrihydrite (15, 16). It also resembles the digestion product of dietary Fe^3+^ in the small bowel (17). NanoFe^3+^ has potential advantages as a novel oral iron supplement. It is absorbed whole into duodenal enterocytes and then readily broken down intralysosomally (17). Soluble iron that is released from the nanostructure in this fashion can be utilized systemically, being readily incorporated into hemoglobin (Hb) in both rats (14) and iron-deficient human volunteers (unpublished results). Notably, the nanoFe^3+^ preparation appears safe compared to soluble forms of iron, as it is not toxic to gut epithelial cells and does not influence the commensal flora negatively (unpublished results). Given the above, as well as current interest in nanoparticle handling by the gastrointestinal tract, a detailed understanding of the absorption and metabolism of nanoFe^3+^ is required.

Dietary nonheme iron in the intestinal lumen is, predominantly, in the Fe^3+^ form (18–20). Prior to intestinal uptake, Fe^3+^ is widely considered to undergo reduction to Fe^2+^, a reaction that is thought to be catalyzed by brush border ferrireductases, such as duodenal cytochrome *b* (Dcytb; refs. 21, 22), although luminal ascorbate may also be involved (23). The reduced iron is then transferred across the brush border membrane and into enterocytes by an Fe^2+^ transporter, namely solute carrier family 11, member 2 and also termed proton-coupled divalent metal ion transporter 1 (DMT-1; refs. 24, 25). Once inside the cell, iron can be stored within intracellular ferritin (26) or, if there are systemic requirements, it can be transported across the basolateral membrane through ferroportin-1 (Fpn; refs. 21, 27, 28). To date, Fpn is the only identified mammalian iron export protein (29). Intestine-specific Fpn-knockout (KO) mice develop severe systemic iron deficiency and show iron accumulation in enterocytes (30).

Intestinal iron efflux through Fpn is tightly regulated at the systemic level by hepcidin, a peptide “master regulator” of iron homeostasis that is encoded by the *Hamp* gene (31). It is produced predominantly by hepatocytes and secreted into the circulation (31), where it binds to cell surface Fpn, causing the iron export protein to be internalized and degraded (32). As such, hepcidin regulates intestinal iron absorption: hepcidin production is increased when the body is iron replete, such that dietary iron absorption is reduced, and is conversely decreased when the body is iron deplete, such that iron absorption is increased (33–36).

The aim of the current study was to investigate whether the absorption of iron from nanoFe^3+^ is mediated basolaterally by Fpn as it appears to be for soluble forms of iron that are acquired by the enterocyte. To address this, we made use of the intestine-specific Fpn-KO mouse. Our results show that systemic absorption of iron from nanoFe^3+^ is under normal Fpn-dependent iron homeostasis.

## MATERIALS AND METHODS

### Iron materials

Ferrous sulfate heptahydrate (FeSO_4_) was purchased from Sigma-Aldrich (Gillingham, Dorset, UK). Ferric citrate monohydrate was purchased from Sigma-Aldrich (Sydney, Australia). Ferric nitrilotriacetate chelate (FeNTA_2_) was produced by mixing an acidified solution of FeCl_3_ (10 mM) with an NTA solution to achieve a molar ratio of Fe:NTA of 1:2. The pH of the final solution was adjusted to 7.4 with NaOH. NanoFe^3+^ was prepared according to the protocol by Powell *et al.* (14). Briefly, an acidic concentrated stock solution of FeCl_3_ was added to a solution containing tartaric acid and adipic acid in 0.9% (w/v) of electrolyte (KCl) to achieve a molar ratio of Fe:tartaric acid:adipic acid in the final suspension of 2:1:1 and [Fe] = 40 mM. The initial pH of the mixture was always below 2.0 and the Fe was fully soluble. The pH was then slowly increased by dropwise addition of a concentrated solution of NaOH until *ca.* pH 7.4. The entire mixture was then oven-dried at 45°C for a minimum of 24 h.

### Rodent diets

All diets were prepared by Specialty Feeds (Glenn Forest, WA, Australia) and supplied in a powdered form. Other than varying the amount and form of the iron added, the diets were equivalent and conformed to AIN-93G purified rodent diet (Supplemental Table S1 and ref. 37). The iron materials used to supplement the rodent diet were FeSO_4_, Fe^3+^ citrate, and nanoFe^3+^ as defined above. The total iron content of the test diets was determined by inductively coupled plasma optical emission spectrometry (ICP-OES; JY2000, Horiba-Jobin, Stanmore, UK) at 259.94 nm following digestion with concentrated nitric acid at 37°C for 4 d, followed by 16 h at 70°C, and then diluted (1:5) with UHP water. ICP-OES calibration was with sample-based standards (the sample matrix used was that of the Fe-deficient diet similarly digested and diluted) which were spiked with iron ranging from 0 to 18 μM, in a similar fashion to previous work (38).

### Animals

Mice carrying the floxed Fpn (*Fpn*^*flox/flox*^) allele were bred with *vil-Cre-ER*^*T2*^ mice, which carry a tamoxifen-inducible, intestine-specific Cre recombinase gene (30). Mice with intestine-specific deletion of Fpn (here referred to as Fpn KO) were produced by injecting the resulting *vil-Cre-ER*^*T2*^*/Fpn*^*flox/flox*^ mice subcutaneously with tamoxifen (0.075 mg/g body weight) once daily for 3 d starting at 28 d of age. Littermate wild-type (WT) control mice were also injected with tamoxifen 1×/d for 3 d starting at 28 d of age. Lack of Fpn expression in intestinal enterocytes taken from tamoxifen-injected mice was confirmed by Western blotting (Supplemental Fig. S1). This study was carried out in strict accordance with the Australian Code of Practice for the Care and Use of Animals for Scientific Purposes. All animal procedures were approved by the Queensland Institute of Medical Research Berghofer Medical Research Institute Animal Ethics Committee (registration number A0192-609M). All surgery was performed under anesthesia, and all efforts were made to minimize suffering.

### Feeding study design and tissue collection

WT and Fpn-KO mice were allocated to the different diet groups (*n*=4–8 mice/set) as outlined in Supplemental Fig. S2. All animals were housed individually and had unlimited access to food and deionized water throughout the study. Three-week-old mice were fed an iron-deficient diet for 4 wk (*i.e.*, 0–4 wk=iron-depletion period) to induce iron deficiency. Administration of the test diets was to the two study groups and comprised the iron-deficient diet supplemented with *ca.* 20 mg iron/kg_diet_ as FeSO_4_ or nanoFe^3+^. This began at the end of week 4 and lasted a further 4 wk (*i.e.*, 4–8 wk; Supplemental Fig. S2). Two further sets of mice (one Fpn KO and the other WT) remained on the iron deficient diet throughout the study (here named Fe-deficient group). Similarly, two control sets of animals (one Fpn KO and the other WT) were fed an iron sufficient diet, with *ca.* 35 mg iron/kg_diet_ as Fe^3+^ citrate monohydrate to conform with AIN-93G diet formulation (37), for the entire 8 wk study period (*i.e.*, from 0 to 8 wk), and this is termed the Fe-sufficient group.

Blood samples were collected from each animal at the end of wk 4 (*i.e.*, after iron depletion for the study groups and Fe-deficient group), wk 5 (1 wk into the iron repletion period for the study groups) and wk 6 (2 wk into the iron repletion period for the study groups) by piercing a facial vein. Approximately 25 μl of blood was collected into EDTA tubes at each time point. After the iron repletion period of the study groups (*i.e.*, end of wk 8), the mice were anesthetized with a single intraperitoneal injection of xylazine (10 mg/kg) and ketamine (200 mg/kg) and euthanized by exsanguination, and blood, liver, spleen, and duodenal tissues were collected. Liver and spleen samples were diced prior to snap-freezing in liquid nitrogen. The proximal section of the small intestine (∼2 cm) was cut open lengthwise, washed in ice-cold PBS, and transferred into 5 ml of enterocyte-isolating solution consisting of 15 μM EDTA, protease inhibitor (Complete; Roche Molecular Biochemicals, Basel, Switzerland) and 5 μM phenylmethylsulfonyl fluoride (PMSF) in PBS. Samples were then gently inverted for 30 min at 4°C. The detached cells were centrifuged (1000 *g*, 4°C, 5 min) and washed twice with ice-cold PBS prior to snap-freezing in liquid nitrogen.

### Measurement of iron absorption using duodenal loops

Duodenal loops were used to assess iron absorption from iron compounds directly introduced into the duodenum (pylorus to the ligament of Treitz; ref. 39). WT and Fpn-KO mice (8–12 wk old) were maintained on an iron-deficient diet for 1 wk prior to the intestinal loop procedure to ensure that the enterocytes were minimally loaded with dietary iron. Mice were anesthetized (200 mg/kg ketamine and 10 mg/kg xylazine), and a midline incision was made in the abdomen. After the duodenum was exposed, two ligatures were tied immediately after the pylorus, and a third was tied immediately before the ligament of Treitz. Special care was taken to prevent ligation of blood vessels. Two incisions were made in the duodenum, one proximal to the first ligature and the other distal to the third ligature. A cannula was inserted through the proximal incision, and the first ligature was tightened to hold the cannula in place. The exposed intestinal segment was flushed with *ca.* 5 ml of saline (prewarmed to 37°C) injected through the cannula. After flushing the exposed segment, the third ligature at the distal end of the duodenum was tightened. The loop was infused with 100 μl of solution containing 500 μM iron as either FeNTA_2_ (soluble iron control; *n*=7 WT and *n*=7 Fpn KO) or nanoFe^3+^ (*n*=10 WT and *n*=9 Fpn KO) in 125 mM NaCl, 3.5 mM KCl, and 16 mM HEPES, pH 7.5 (iron phase distribution in this solution confirmed that FeNTA_2_ was mainly soluble and nanoFe^3+^ was mainly nanoparticulate; Supplemental Fig. S3). Approximately 100 μl of saline was then infused through the cannula to flush any residual test solution into the intestinal lumen. The second ligature was tightened to seal the test solution inside the intestinal loop and the cannula removed. The abdomen was then covered with damp gauze, which was continually moistened with prewarmed saline to prevent drying. Blood was collected by heart puncture 30 min after infusion, and the animals were killed by cervical dislocation.

### Analysis of blood parameters and tissue iron stores

The Hb concentration in the blood samples was determined with a Coulter Ac·T diff Analyzer (Beckman Coulter Australia, Sydney, NSW, Australia) according to the manufacturer's protocol. Histochemical staining for iron in paraffin-embedded duodenal, liver, and spleen sections was carried out with Perls' Prussian blue (40) using hematoxylin-eosin as a counterstain. Serum iron levels were determined colorimetrically using a ferrozine-based iron assay kit (Pointe Scientific Inc., Canton, MI, USA) following the manufacturer's instructions. Liver and spleen iron levels were also determined colorimetrically using a modification of the ferrozine-based assay described by Rebouche *et al.* (41). Briefly, liver and spleen samples were wrapped in foil and dried overnight in an oven at 110°C. Samples were mixed with a solution containing 3 M hydrochloric acid and 0.6 M trichloroacetic acid. Calibration standards were prepared in the range 2–100 μg Fe/ml. Tissues and standards were incubated at 65°C for 20 h, then vortexed and centrifuged (10,000 *g*, 5 min). Two parts of chromogen solution (0.508 mM ferrozine, 1.5 M sodium acetate, and 1.5% v/v thioglycolic acid in H_2_O_UHP_) were mixed with 1 part standard/tissue supernatant, and the absorbance was read at 595 nm following 30 min incubation at room temperature.

### Analysis of gene expression

RNA was extracted from snap-frozen liver and duodenal tissue samples using TRIzol reagent (Invitrogen, Melbourne, VIC, Australia) as per the manufacturer's instructions. The RNA (500 ng) was then used for cDNA synthesis using Moloney murine leukemia virus reverse transcriptase (Invitrogen) and an oligo-dT primer as per the manufacturer's instructions. Gene expression was determined by quantitative real-time polymerase chain reaction (rtPCR) as described previously (42).

The primers used were as follows: *Hamp1*, forward CCTGAGCAGCACCACCTATC, reverse TGCAACAGATACCACACTGGG; *Slc11a2*, forward TCATACCCATCCTCACGTTCAC, reverse GGTCAAATAAGCCACGCTAACC; *HPRT*, forward ATGATCAGTCAACGGGGGAC, reverse TTGGGGCTGTACTGCTTAAC.

### Western blotting

Protein was extracted from isolated enterocytes, and expression of Fpn was assessed by Western blotting as described previously (43).

### Statistical analysis

Unless otherwise stated, all values are expressed as means ± sd. Statistical differences between the Hb values for each group of mice at the various time points on a specific diet were determined with repeated measures 2-way analysis of variance (ANOVA) with the Bonferroni correction to account for multiple comparisons. Statistical differences between hepatic and splenic tissue iron levels and gene expression levels between groups of mice on the various diets were determined with 1-way ANOVA with the Bonferroni correction. Statistical differences between the serum iron levels from the duodenal loop study were determined by unpaired *t* test. The level of significance was set to *P* < 0.05. Statistical analysis were performed with GraphPad Prism 6 (GraphPad Software, San Diego, CA, USA).

## RESULTS

### Dietary nanoFe^3+^ is absorbed *vi*a a Fpn-dependent pathway

**Figure 1.**
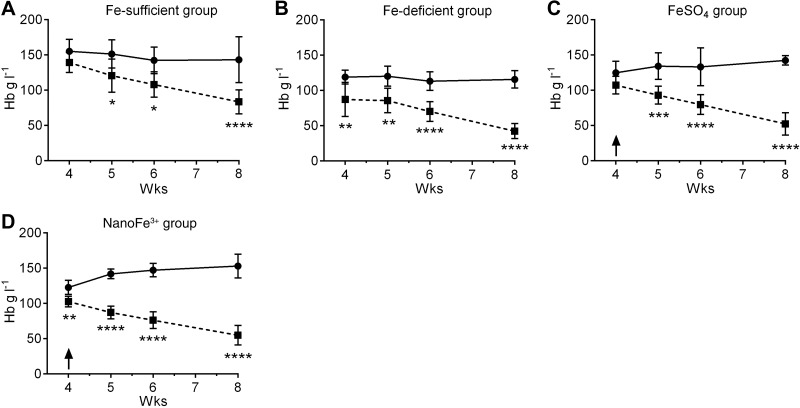
Hb levels in mice during dietary iron repletion. Hb values for WT (solid trace) and Fpn KO (dashed trace) mice in the control Fe-sufficient (*i.e.*, non-iron-depleted) group (*A*), the Fe-deficient group (*B*), the FeSO_4_-supplemented group (*C*), and the nanoFe^3+^-supplemented group (*D*) after iron depletion (study wk 4), 1 wk into the iron repletion period (study wk 5), 2 wk into the iron repletion period (study wk 6), and at the end of the iron repletion period (study wk 8) as per study outline presented in Supplemental Fig. S2. Arrows (*C*, *D*) indicate the start of iron repletion with FeSO_4_- or nanoFe^3+^-supplemented diets, respectively. Values are means ± sd. Numbers in each group are as follows: Fe-sufficient, *n* = 6 WT and *n* = 8 Fpn KO; Fe-deficient, *n* = 6 WT and *n* = 6 Fpn KO; FeSO_4_, *n* = 4 WT and *n* = 9 Fpn KO; nanoFe^3+^, *n* = 8 WT and *n* = 6 Fpn KO. *P ≤ 0.01, **P ≤ 0.007, ***P ≤ 0.0002, ****P < 0.0001 *vs*. Fpn-KO mice.

Hb levels in the Fpn-KO mice maintained on the iron-sufficient diet (Fe-sufficient group) were lower than the Hb levels of the WT mice maintained on the same diet, being significant from wk 5 onward (*P*≤0.03; **Fig. 1*A***). Similarly, Fpn-KO mice maintained on the iron-deficient diet (Fe-deficient group) had lower Hb compared to WT mice on the same diet, and this was statistically significant from wk 4 onward (*P*<0.003; Fig. 1*B*). Fpn-KO mice on both iron-supplemented test diets (*i.e.*, FeSO_4_ and nanoFe^3+^ groups) also had similar and significantly lower Hb levels throughout the Fe repletion period compared to the WT mice (*P*≤0.0002 from wk 5 for FeSO_4_ and *P*≤0.007 from wk 4 for nanoFe^3+^; Fig. 1*C*, *D*).

The body weights of the animals for each dietary group are presented in Supplemental Table S2 alongside the baseline and final Hb values of Fig. 1. There were no significant differences in the body weight of the animals in any diet group at the end of the study. There were no statistically significant differences between final Hb levels of the WT mice supplemented with nanoFe^3+^ in comparison to WT mice supplemented with FeSO_4_ or WT mice in the control Fe-sufficient group. However, Hb levels at the end of the study in the nanoFe^3+^-supplemented WT mice were significantly higher than for WT mice of the Fe-deficient group (*P*=0.005; Supplemental Table S2). For the Fpn-KO mice, Hb levels at the end of the study in the nanoFe^3+^ group were similar to the Fe-deficient group and the FeSO_4_ group, but were lower for these 3 groups than the levels in the Fe-sufficient group (*P*=0.06 for nanoFe^3+^, *P*=0.01 for FeSO_4_, and *P*=0.001 for Fe-deficient; Supplemental Table S2).

Throughout, Hb levels of Fpn-KO mice were significantly lower than for WT counterparts (all *P*≤0.0001 by wk 8; Fig. 1).

### Tissue iron levels

**Figure 2.**
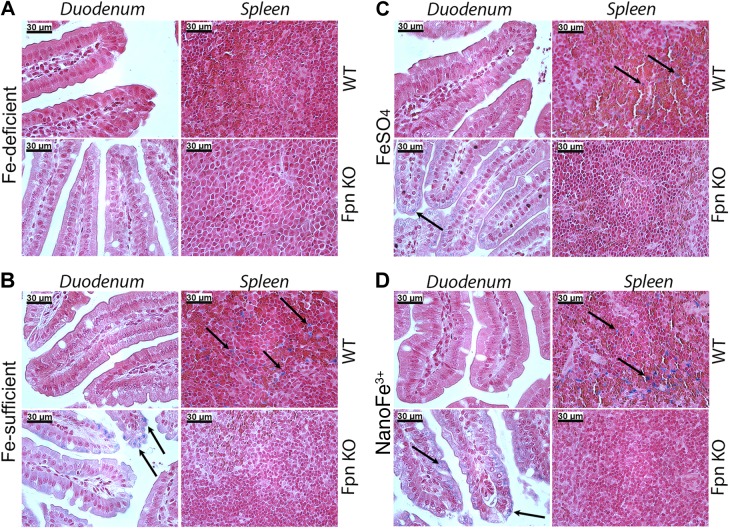
Tissue Fe distribution. Representative images of Perls' Prussian blue staining of duodenum and spleen of WT and Fpn-KO mice in the Fe-deficient (*A*), control Fe-sufficient (*B*), FeSO_4_-supplemented (*C*), or nanoFe^3+^-supplemented (*D*) groups, defined as per study outline presented in Supplemental Fig. S2. Arrows indicate example locations of iron staining in the tissues. Scale bars = 30 μm.

The levels of iron in the duodenum, spleen, and liver of animals maintained on diets containing different forms of iron were assessed qualitatively by Perls' Prussian blue staining and quantitatively (only for the spleen and liver) using a colorimetric assay. There was no stainable iron in the small intestinal enterocytes of WT or Fpn-KO mice fed the iron-deficient diet throughout the study (**Fig. 2*A***). Similarly, there was no accumulation of iron in the spleen (Fig. 2*A*) or the liver in these mice (Supplemental Fig. S4). In the three other groups (*i.e.*, both iron-supplemented groups and the non-iron-depleted control group), no stainable iron was seen in the small intestinal enterocytes of WT mice, but distinct iron accumulation was observed in enterocytes of Fpn-KO mice (Fig. 2*B–D*). Conversely, iron staining was detected in the spleens of WT mice but not in the spleen of Fpn-KO mice (Fig. 2*B–D*). No detectable iron staining was observed in the liver of either WT or Fpn-KO animals from any group (Supplemental Fig. S3).

**Figure 3.**
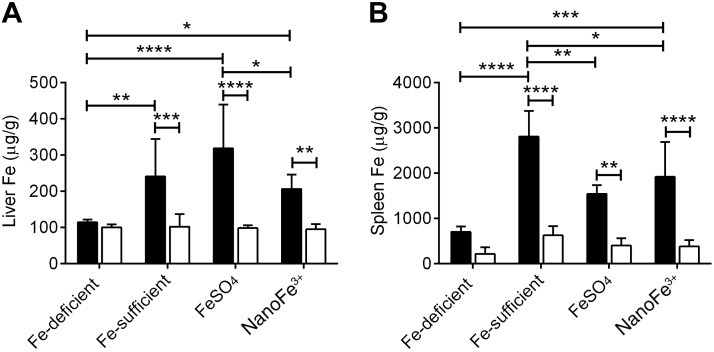
Liver and spleen nonheme iron levels. Hepatic iron (*A*) and splenic iron (*B*) levels of WT (solid bars) and Fpn-KO (open bars) mice of the control Fe-sufficient (*i.e.*, non-iron-depleted) group, Fe-deficient group, FeSO_4_-supplemented group, and nanoFe^3+^-supplemented group. Data represent means ± sd. Numbers in each group are as follows: Fe-sufficient, *n* = 6 WT and *n* = 8 Fpn KO; Fe-deficient, *n* = 6 WT and *n* = 6 Fpn KO; FeSO_4_, *n* = 4 WT and *n* = 9 Fpn KO; nanoFe^3+^, *n* = 8 WT and *n* = 6 Fpn KO. *P ≤ 0.04, **P ≤ 0.006, ***P ≤ 0.0009, ****P < 0.0001.

Quantitation of iron levels in the liver of WT animals showed that both the FeSO_4_- and the nanoFe^3+^-supplemented groups were able to increase hepatic Fe stores to levels similar to those observed in the non-iron-depleted control group (**Fig. 3**). Corresponding increases in spleen iron also occurred in both the FeSO_4_- and nanoFe^3+^-supplemented groups, although the levels did not reach that of control mice. In contrast, both liver and spleen iron levels were consistently low in the Fpn-KO mice in all the diet groups and were significantly lower than the levels in the corresponding WT animals (*P*≤0.006; Fig. 3). Spleen iron levels were an order of magnitude higher than liver iron levels for all diet groups (Fig. 3).

### Tissue expression of Fe-related genes

**Figure 4.**
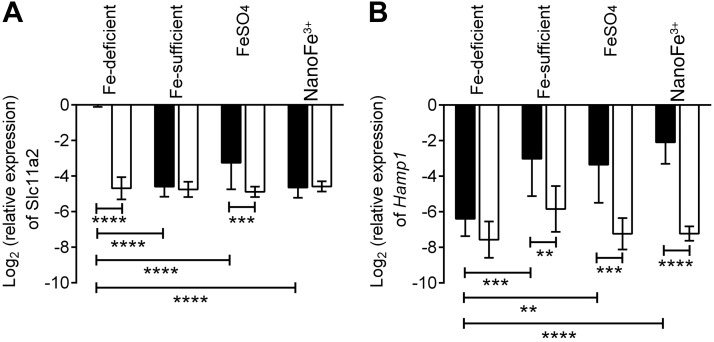
Expression of *Slc11a2* mRNA (*A*) in isolated enterocytes and *Hamp1* (*B*) in the liver. Data shown for WT (solid bars) and Fpn-KO (open bars) mice in the control Fe-sufficient (*i.e.*, non-iron-depleted) group, Fe-deficient group, FeSO_4_-supplemented group, and nanoFe^3+^-supplemented group. Values are means ± sd of the log_2_-transformed gene expression in relation to the housekeeping gene *HPRT*. Numbers in each group are as follows: Fe-sufficient, *n* = 6 WT and *n* = 8 Fpn KO; Fe-deficient, *n* = 6 WT and *n* = 6 Fpn KO; FeSO_4_, *n* = 4 WT and *n* = 9 Fpn KO; nanoFe^3+^, *n* = 8 WT and *n* = 6 Fpn KO. **P ≤ 0.007, ***P ≤ 0.0006, ****P < 0.0001.

To investigate how iron derived from nanoFe^3+^ influenced intestinal and systemic iron homeostasis, we examined the enterocyte mRNA expression of *Slc11a2*, the apical transporter of ferrous iron in enterocytes (25), and hepatic hepcidin (*Hamp1*), the master regulator of iron homeostasis (31). In WT mice, *Slc11a2* expression in the duodenum was significantly higher (*P*<0.0001) in the iron-deficient group than in the mice fed the iron-supplemented diets (FeSO_4_ and nanoFe^3+^ groups) or the non-iron-depleted controls (Fe-sufficient group) (**Fig. 4*A***). The relative expression of *Hamp1* mRNA in the liver was significantly higher in both the WT mice administered the iron-supplemented diets or the non-iron-depleted controls compared to the WT mice on the iron-deficient diet (*P*≤0.007; Fig. 4*B*). All Fpn-KO mice had very little expression of either of the iron-regulating genes, irrespective of diet group.

### Iron transport from nanoFe^3+^ in isolated duodenal loops *in vivo*

**Figure 5.**
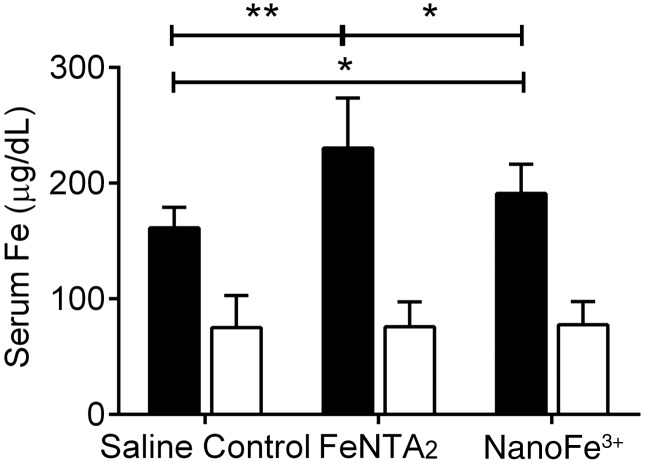
Serum iron levels in Fpn-KO (open bars) and WT (solid bars) mice following 30 min exposure of duodenal loops to iron. Duodenal loops in male mice (8–12 wk old) were infused with either saline (control), 500 μM iron as FeNTA_2,_ or 500 μM iron as nanoFe^3+^. Data represent means ± sd. Numbers in each group are as follows: saline control, *n* = 6 WT and *n* = 7 Fpn KO; FeNTA_2_, *n* = 7 WT and *n* = 7 Fpn KO; nanoFe^3+^, *n* = 10 WT and *n* = 9 Fpn KO. *P ≤ 0.04, **P = 0.007.

Duodenal loops were used to assess the direct transfer of iron, derived from nanoFe^3+^ of the lumen, into the blood circulation in WT and Fpn-KO mice. Infusing the duodenal loops with nanoFe^3+^ provides additional information on the uptake of Fe from the intact nanoparticulate material, that is, bypassing the stomach, unlike with feeding experiments. Serum iron levels were compared to a soluble Fe^3+^ control (FeNTA_2_) and a saline control (no iron). All Fpn-KO animals had significantly lower serum iron levels than WT mice in the same diet group (*P*<0.0001). In WT mice, serum iron levels increased 30 min following infusion with nanoFe^3+^ (*P*=0.04) and more so with FeNTA_2_ (*P*=0.007) (**Fig. 5**). In contrast, there was no difference in serum iron levels of Fpn-KO mice 30 min following infusion with either iron preparation in comparison to the saline control (Fig. 5).

## DISCUSSION

We have previously shown that nanoFe^3+^ crosses the apical surface of Caco-2 cells *via* an endocytic pathway before being dissolved in endosomes or lysosomes inside the cell (17). However, the mechanism by which the iron derived from nanoFe^3+^ crosses the basolateral membrane is unknown. Since Fpn is the only known mammalian iron export protein (29), we investigated the absorption of nanoFe^3+^ in intestine-specific Fpn-KO mice and littermate controls. We found that, like other forms of iron in the intestine, nanoFe^3+^ was ineffective at repleting Hb levels in Fpn-KO mice. Iron was retained in the enterocyte of Fpn-KO animals regardless of whether it was soluble iron or nanoFe^3+^, consistent with Fpn being the common enterocyte exporter for iron irrespective of its luminal form. The low levels of iron in the liver and spleen of Fpn-KO mice provide further evidence that iron derived from nanoFe^3+^ cannot bypass the Fpn-mediated efflux mechanism that is used by soluble luminal iron sources.

We further investigated the absorption of nanoFe^3+^ using intestinal loops in which the iron compound was administered directly into the duodenum of WT and Fpn-KO animals, thereby bypassing the stomach. Although serum iron levels increased significantly with the soluble (FeNTA_2_) and nanoFe^3+^ infusions compared to saline, the increase with nanoFe^3+^ was significantly lower (*P*=0.03) than with FeNTA_2_, despite nanoFe^3+^ being as effective as ferrous sulfate at increasing Hb levels during the dietary intervention study. The difference is not related to the choice of positive controls for the two experiments, as ferrous sulfate is certainly as well absorbed as FeNTA_2_ (44–46). Instead, the data show that either the passage of nanoFe^3+^ through the stomach is required for efficient absorption, or nanoFe^3+^ absorption is slower than that of soluble iron, because the direct uptake of whole nanoparticles requires their endosomal/lysosomal breakdown prior to systemic release of iron (17). Indeed, we suggest that the *ca.* 30 μg/dl difference in serum Fe levels between the FeNTA_2_- and nanoFe^3+^-treated WT animals is mainly due to differences in the absorption kinetics of the two materials. Studies have shown that the rate of absorption of Fe from ferritin, which contains an Fe^3+^ oxohydroxide core very similar to nanoFe^3+^ and is taken up by clathrin-dependent endocytosis (47–49), is slower than that of soluble Fe in the rat intestine (50). Moreover, our own observations in human volunteers comparing nanoFe^3+^ to FeSO_4_ support this difference in kinetics (unpublished results).

Despite these potential differences in the rate of iron absorption by the intestine, the overall response of the body to nanoFe^3+^ appears to be almost identical to that of FeSO_4_. The Hb repletion data in the WT mice demonstrate that iron from nanoFe^3+^ is as efficient at correcting diet-induced iron deficiency anemia as FeSO_4_. Gene expression analysis showed that *Slc11a2* mRNA levels in enterocytes decreased to similar levels in response to iron repletion by either nanoFe^3+^ or soluble Fe. Interestingly, the Fpn-KO animals in all the diet groups expressed very low levels of *Slc11a2* even though they were iron deficient. This is likely due to the accumulation of iron in Fpn-KO enterocytes, as seen in Fig. 2, which leads to the destabilization of *Slc11a2* mRNA *via* the IRP/IRE system (51). Indeed, similar down-regulation of *Slc11a2* levels was observed in sla mice, which develop iron-loaded enterocytes due to a deletion in the gene encoding the ferroxidase hephaestin (52). Liver *Hamp1* mRNA expression also responded similarly to iron repletion irrespective of the iron source. WT animals in the iron-supplemented groups showed significantly elevated *Hamp1* expression in the liver when compared to iron-deficient animals.

Taken together, our data clearly demonstrate the effectiveness of nanoFe^3+^ as a dietary supplement, consistent with iron repletion studies in rats (14). The relatively slow release of iron from nanoFe^3+^, with a consequent reduced rate of absorption, may be an advantage in terms of preventing the generation of nontransferrin bound Fe that can be observed following absorption of therapeutic doses of soluble iron (53–56). Notably, the iron derived from nanoFe^3+^ does not circumvent systemic iron regulatory mechanisms, as the intestinal efflux of iron following enterocyte uptake of nanoFe^3+^ is ferroportin mediated in a similar manner to that of soluble iron. This implies that iron derived from nanoFe^3+^ joins the common enterocyte labile Fe pool, at some point, following the direct brush border uptake of nanoFe^3+^.

## Supplementary Material

Supplemental Data
