# Supplementary material for: Ferroportin mediates the intestinal absorption of iron from a nanoparticulate ferritin core mimetic in mice
Source: FASEB J. 2014 Aug;28(8):3671–8. doi: 10.1096/fj.14-251520 (PMC4101650; doi:10.1096/fj.14-251520)
Supplement: Supplemental Data [file supp_28_8_3671__index.html]

Ferroportin mediates the intestinal absorption of iron from a nanoparticulate ferritin core mimetic in mice — Ferroportin mediates the intestinal absorption of iron from a nanoparticulate ferritin core mimetic in mice — Supplemental Data 

# Ferroportin mediates the intestinal absorption of iron from a nanoparticulate ferritin core mimetic in mice

## Supplemental Data

**Files in this Data Supplement:**

- Supplemental Data - (*14-251520SuppData.zip; compressed file 609 KB*)
