# Supplementary material for: Ferroportin mediates the intestinal absorption of iron from a nanoparticulate ferritin core mimetic in mice
Source: FASEB J. 2014 Aug;28(8):3671–8. doi: 10.1096/fj.14-251520 (PMC4101650; doi:10.1096/fj.14-251520)
Supplement: Supplemental Data [file supp_fj.14-251520_14-251520SuppData.zip › Supplementary Tables S1 & S2.pdf]

Table S1: Composition of basal (iron deficient) diet (1). Iron sufficient, $FeSO_4$ and Nano Fe(III) diets corresponded to the same basal diet with added iron as Fe(III) citrate (~35 mg Fe/kg_{diet}), Fe(II) sulfate (~20 mg Fe/kg_{diet}) or tartrate-modified Fe(III) poly oxo-hydroxide (~20 mg Fe/kg_{diet}) respectively.

Ingredients	Content (g/100g)	Ingredients	Content (g/100g)
**Sucrose**	10.00	**Calcium carbonate**	1.10
**Casein (Acid)**	20.00	**Salt (fine sodium chloride)**	0.25
**Canola oil**	7.00	**Potassium phosphate monobasic**	0.69
**Cellulose**	5.00	**Potassium sulfate**	0.16
**Wheat starch**	40.60	**Potassium citrate**	0.25
**Dextrinised starch**	13.20	**Magnesium oxide**	0.05
**Di methionine**	0.30	**AIN-93 Vitamins**	1.00
**AIN-93 Trace minerals no iron**	0.14	**Choline chloride 75% w/w**	0.25

1. Reeves, P. G., Nielsen, F. H., and Fahey, G. C., Jr. (1993) AIN-93 purified diets for laboratory rodents: final report of the American Institute of Nutrition ad hoc writing committee on the reformulation of the AIN-76A rodent diet. *J Nutr* **123**, 1939-1951

Table S2: Body weight and baseline and final haemoglobin levels of WT and Fpn KO mice on different test diets for 4 weeks. Data are shown as mean ± SD.

	**Fortification level** [(1)] (mg Fe/kg_{diet})	**Mice** (N)	**Final bodyweight (g)** *Bodyweight gain (g/4 weeks)*[(2)]	**Baseline Hb (i.e.week 4)** (g L^{-1})[(3)]	**Final Hb (i.e. week 8)** (g L^{-1})[(3)]
WT					
Fe-deficient	1.0 ± 0.2	6	**22.5 ± 1.6** *2.7 ± 1.4*	118.8 ± 9.9 [a]	115.7 ± 12.5 [a]
Control Fe-sufficient	43.1 ± 0.2	6	**25.8 ± 2.4** *1.4 ± 1.4*	155 ± 16.9 [b]	143.0 ± 32.7 [a b]
$FeSO_4$	19.4 ± 0.4	4	**24.7 ± 2.6** *3.1 ± 1.7*	124.8 ± 16.1 [a]	142.3 ± 6.7 [a b]
Nano Fe(III)	22.9 ± 0.1	8	**24.2 ± 1.7** *2.8 ± 1.0*	122.6 ± 7.7 [a]	152.9 ± 16.8 [b]
Fpn KO					
Fe-deficient	1.0 ± 0.2	6	**22.1 ± 2.9** *1.8 ± 1.6*	87.0 ± 24.3 [a]	42.2 ± 10.7 [a]
Control Fe-sufficient	43.1 ± 0.2	6	**24.6 ± 2.2** *2.0 ± 1.2*	139.1 ± 14.2 [b]	83.4 ± 17.0 [b c]
$FeSO_4$	19.4 ± 0.4	9	**23.2 ± 2.4** *1.6 ± 1.7*	107.1 ± 12.6 [a]	52.0 ± 16.0 [a]
Nano Fe(III)	22.9 ± 0.1	6	**24.0 ± 1.6** *0.8 ± 1.5*	102.5 ± 10.1 [a]	54.8 ± 13.9 [a c]

(1) Iron content in the test diets as determined by inductively coupled plasma optical emission spectrometry (ICP-OES). **(2)** Bodyweight gain corresponds to weight change between study week 4 and 8. **(3)** For each strain within a column, means with different superscript letters (a,b,c) are significantly different. The haemoglobin values are the same (but numerical) data of that graphed in Figure 1 for baseline and final values.
