# Supplementary material for: Ferroportin mediates the intestinal absorption of iron from a nanoparticulate ferritin core mimetic in mice
Source: FASEB J. 2014 Aug;28(8):3671–8. doi: 10.1096/fj.14-251520 (PMC4101650; doi:10.1096/fj.14-251520)
Supplement: Supplemental Data [file supp_fj.14-251520_14-251520SuppData.zip › Supplementary Figures S1 & S2.pdf]

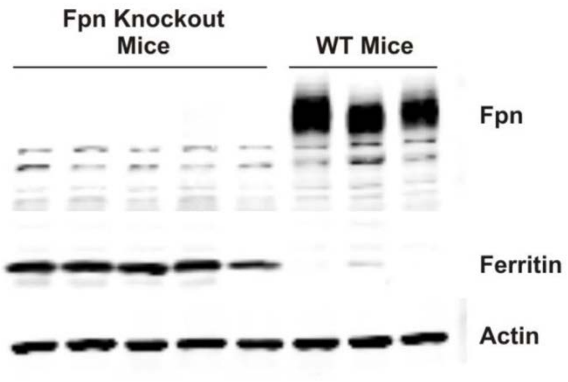

Figure S1. Representative western-blot analysis of Fpn and ferritin expression in duodenal extracts from 15 day old intestinal specific Fpn knockout mice and littermate controls. All mice were treated with tamoxifen as described in Materials and Methods.

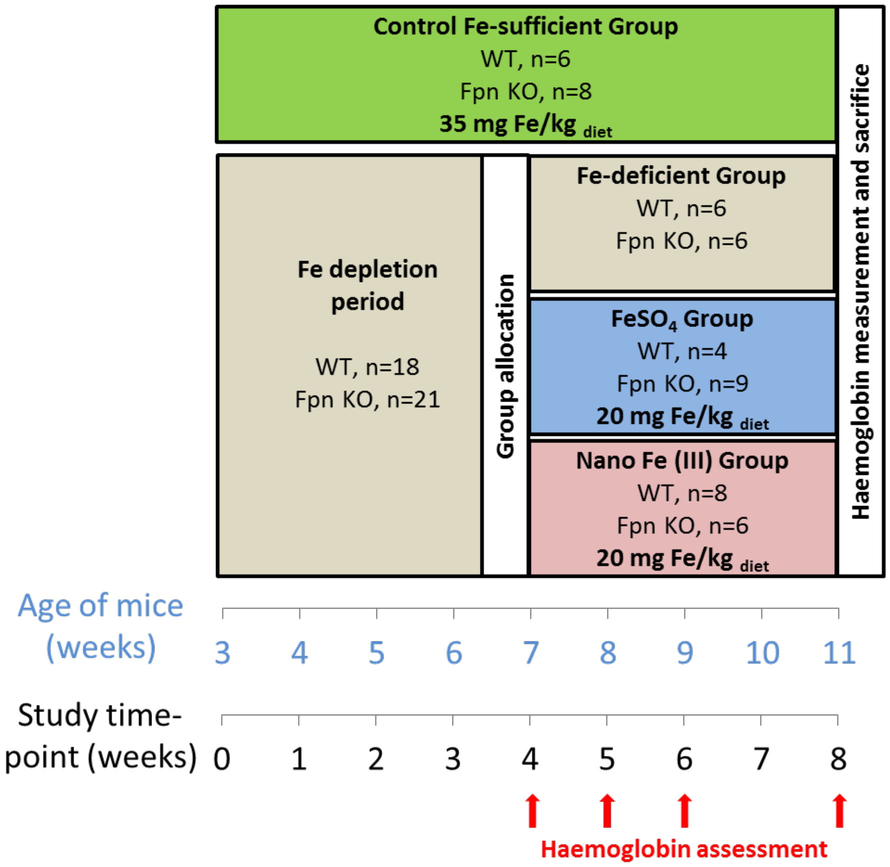

Figure S2. Schematic outline of study design. WT (n=18) and Fpn KO (n=21) mice were weaned onto a low iron diet (iron depletion period) at three weeks of age (study week 0) and were maintained on this Fe-deficient diet for 4 weeks (until study week 4). Haemoglobin levels were assessed post iron depletion (i.e. at study week 4) following which mice were placed on an $FeSO_4$ or Nano Fe(III) supplemented diet for another 4 weeks (i.e. until study week 8). A group of iron deficient animals (n=6 WT and n=6 Fpn KO) continued on the iron deficient diet until the end of the study (i.e. study week 8). For all groups, haemoglobin levels were assessed at weeks 5, 6 and 8, following which the animals were sacrificed and tissues were collected. For the entire 8 weeks duration of the study, a group of animals (n=6 WT and n=8 Fpn KO) were maintained on an iron sufficient diet and assessed at the same time points as the other groups, this group acted as the non-iron depleted control group.
