# Supplementary material for: Ferroportin mediates the intestinal absorption of iron from a nanoparticulate ferritin core mimetic in mice
Source: FASEB J. 2014 Aug;28(8):3671–8. doi: 10.1096/fj.14-251520 (PMC4101650; doi:10.1096/fj.14-251520)
Supplement: Supplemental Data [file supp_fj.14-251520_14-251520SuppData.zip › Supplementary Figure S3 & Methods.pdf]

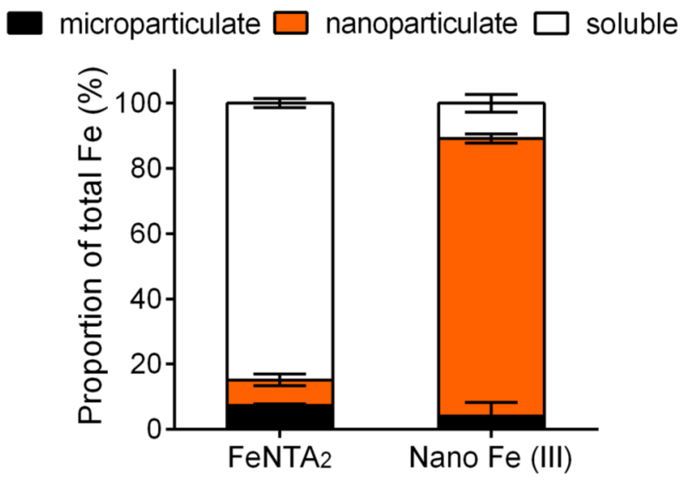


**Figure S3. Phase distribution of the FeNTA2 and Nano Fe(III) in the duodenal loops infusion.** Materials were dispersed in 125mM NaCl, 3.5mM KCl and 16mM HEPES (pH 7.5) at an Fe concentration of 500 µM. Data presented for the fractional percentage of microparticulate (black), nanoparticulate (orange) and soluble Fe (white). Data was collected immediately following homogenisation. Values are mean ± SD (n=2 replicates).

**Supplementary methodology: *Phase distribution*.** Fractionation of the Fe into percentages of nanoparticulate, microparticulate and soluble Fe in the solution used to infuse the duodenal loops was achieved by centrifugation and ultrafiltration. The 500µM preparations of Nano Fe(III) or $FeNTA_2$ were centrifuged (10,000 *xg*, 5 min) and the sediment considered as the microparticulate fraction. In order to isolate the soluble Fe and to distinguish it from nanoparticulate Fe, the supernatant was further ultrafiltered ($M_r$ cut-off 3,000 Da; 10,000 x *g*, 10 min). The Fe content of total, supernatant and ultrafiltrate fractions was determined by inductively-coupled plasma optical emission spectrometry (ICP-OES JY 2000, Horiba Jobin Yvon Ltd., Stanmore, UK) at 259.94 nm. ICP-OES standards and samples were diluted in 0.5 % $HNO_3$ to concentrations in the range 0-1000 ppb. Microparticulate, nanoparticulate and soluble iron were expressed as percentage ± SD in relation to total Fe content as follows:

$$[(\%)\ \text{Fe microparticulate}] = [(\text{Total Fe} - \text{Fe}_{\text{supernatant}})/\text{Total Fe}] \times 100$$

$$[(\%)\ \text{Fe nanoparticulate}] = [(\text{Fe}_{\text{supernatant}} - \text{Fe}_{\text{ultrafiltrate}})/\text{Total Fe}] \times 100$$

$$[(\%)\ \text{Fe soluble}] = [(\text{Fe}_{\text{ultrafiltrate}})/\text{Total Fe}] \times 100$$
