# Supplementary material for: Ferroportin mediates the intestinal absorption of iron from a nanoparticulate ferritin core mimetic in mice
Source: FASEB J. 2014 Aug;28(8):3671–8. doi: 10.1096/fj.14-251520 (PMC4101650; doi:10.1096/fj.14-251520)
Supplement: Supplemental Data [file supp_fj.14-251520_14-251520SuppData.zip › Supplementary Figure S4.pdf]

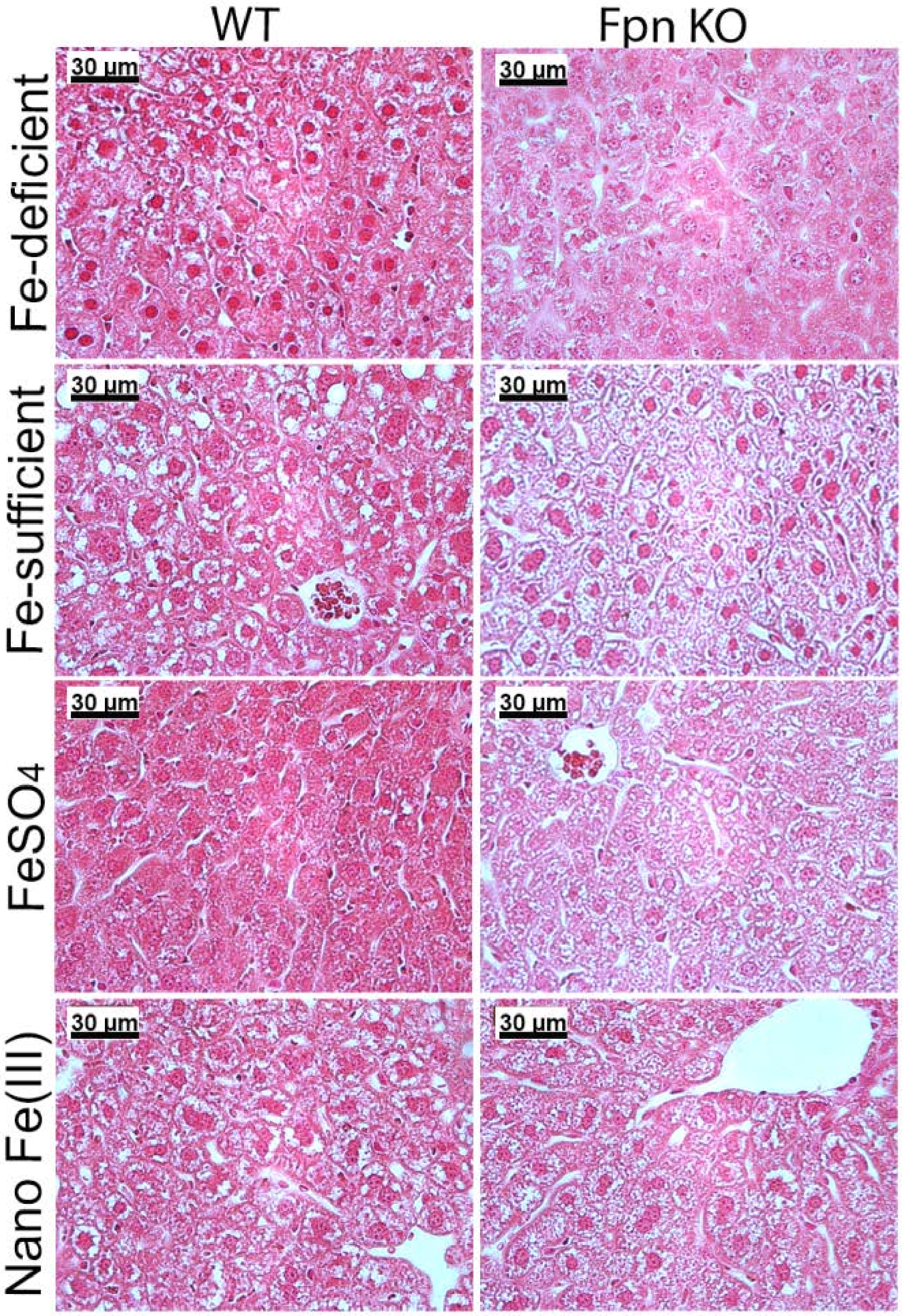


**Figure S4**. **Liver iron distribution.** Perls' Prussian Blue staining in the liver of WT and Fpn KO mice in the Fe-deficient, control Fe-sufficient, $FeSO_4$ or Nano Fe(III) groups defined as per Figure S2. No stainable iron was detected. Scale bars represent 30 µm.
